# SMART: recent updates, new developments and status in 2020

**DOI:** 10.1093/nar/gkaa937

**Published:** 2020-10-26

**Authors:** Ivica Letunic, Supriya Khedkar, Peer Bork

**Affiliations:** biobyte solutions GmbH, Bothestr 142, 69126 Heidelberg, Germany; EMBL, Meyerhofstrasse 1, 69117 Heidelberg, Germany; EMBL, Meyerhofstrasse 1, 69117 Heidelberg, Germany; Max Delbrück Centre for Molecular Medicine, Berlin, Germany; Department of Bioinformatics, Biocenter, University of Würzburg, Würzburg, Germany

## Abstract

SMART (Simple Modular Architecture Research Tool) is a web resource (https://smart.embl.de) for the identification and annotation of protein domains and the analysis of protein domain architectures. SMART version 9 contains manually curated models for more than 1300 protein domains, with a topical set of 68 new models added since our last update article (1). All the new models are for diverse recombinase families and subfamilies and as a set they provide a comprehensive overview of mobile element recombinases namely transposase, integrase, relaxase, resolvase, cas1 casposase and Xer like cellular recombinase. Further updates include the synchronization of the underlying protein databases with UniProt (2), Ensembl (3) and STRING (4), greatly increasing the total number of annotated domains and other protein features available in architecture analysis mode. Furthermore, SMART’s vector-based protein display engine has been extended and updated to use the latest web technologies and the domain architecture analysis components have been optimized to handle the increased number of protein features available.

## INTRODUCTION

Protein domain databases remain important annotation and research tools. The SMART database (5) integrates manually curated hidden Markov models (6,7) for many domains with a powerful web-based interface offering various analysis and visualization tools. Almost 25 years since its inception, it remains popular and widely used by scientists worldwide. Here we summarize the major changes and new features that have been introduced since our last report (1).

## TOPICAL EXPANSION OF DOMAIN COVERAGE

**Figure 1.**
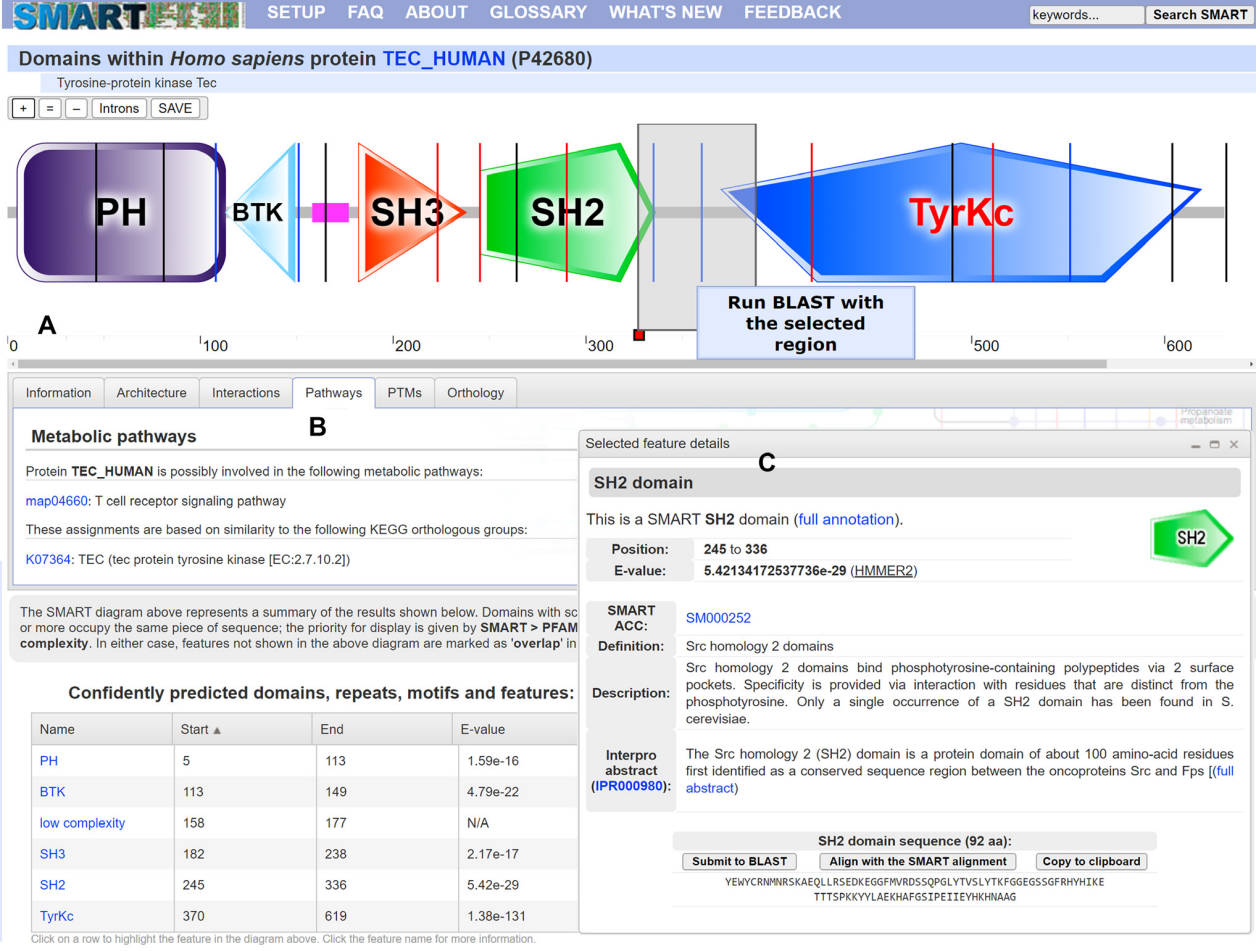
SMART annotation page for protein TEC_HUMAN. (**A**) Protein schematic representations are displayed using an interactive SVG (scalable vector graphics) applet. Schematics are zoomable without quality loss and can be saved as vector (SVG) images. Protein features selected in various data tables are dynamically highlighted directly in the viewer. Using the interactive scale, any protein region can be selected and submitted for further BLAST analysis. (**B**) Tabbed interface collects various sources of external information about the protein analyzed. (**C**) Movable and resizable popup dialog displays the most important bits of information for any selected feature, with links to complete annotation.

SMART was never intended to be exhaustive, and was initially focused on mobile domains, which occur in various contexts while retaining similar function. Nevertheless, it continues to gradually expand its domain coverage with each new release. The current version introduces a topical expansion geared towards a complete coverage of known prokaryotic recombinases, which can be searched together for a report on mobile genetic elements in bacteria and archaebacteria. For this purpose, we bring together a comprehensive set of 68 domains that cover five major recombinase families namely transposase—DDE (8), relaxase—HUH (9), casposase—cas1 (10), (resolvase-) serine and (integrase-) tyrosine recombinase (11). Thirty-one models for DDE subfamilies, 10 for HUH and 1 for tyrosine recombinase were obtained from Pfam (12), a set of 20 tyrosine recombinases sub-families from (13) and six sub-families three belonging to serine, two to HUH and one to Cas1 were newly developed. Based on the association of most of the recombinase sub-families with specific mobile element types or cellular functions, these 68 domains together can be used as seeds for the identification and discrimination of diverse mobile element types namely transposable elements, phages, integrons, conjugative elements (plasmids and Integrative Conjugative Elements—ICE) and casposons. All together, these new domains are present in close to 1.1 million proteins from the current SMART non-redundant database (Figure 1).

## UPDATED PROTEIN DATABASES

The main underlying protein database in SMART combines the complete Uniprot (2) with all stable Ensembl (3) proteomes. The current release contains more than 137 million proteins (a 2.7-fold increase compared to the previous release) from around 537 thousand species, subspecies and strains (a 1.2-fold increase). To reduce the high redundancy that is inherently present in these databases, SMART uses a per-species protein clustering procedure. All the proteins are initially separated into species-specific databases. Each of these databases is clustered separately using the CD-HIT algorithm (14) with a 96% identity cutoff. Longest members of each cluster are used as ‘representatives’, and are the only proteins included in the database, together with all the singletons. This procedure significantly improves the results of all domain architecture queries and brings the domain counts to lower levels, comparable to the genomic mode database described below. The clustering procedure created 9.8 million multi protein clusters containing a total of 25 million proteins.

In addition to the regular protein database described above, SMART offers a ‘genomic’ analysis mode which contains only proteins from completely sequenced genomes. Synchronized with the current STRING version 11 (4), it currently contains >24 million proteins from 5090 complete genomes (477 *Eukaryota*, 4445 *Bacteria* and 168 *Archaea*), which is a 2.5-fold increase in both the number of proteins and genomes.

## UPDATED PROTEIN VISUALIZATION ENGINE

Since the previous SMART release (version 8), all SMART protein schematics (‘bubblograms’) are generated in vector graphics formats (Figure 1). Protein schematics in various list displays (such as domain architecture analysis results) are displayed as dynamically generated inline SVG (Scalable Vector Graphics) images, which seamlessly scale to the users display size, regardless of its resolution. In SMART version 9, we have optimized all image generation routines to support latest web standards.

Version 9 brings a new version of the display applet used in the single protein annotation mode. It uses interactive SVG for the visualization and user interaction, offering the widest browser support. Protein schematics can be zoomed in to any level without quality loss, and saved as vector graphics for easy inclusion in user documents or publications.

Like the previous versions, the new SMART protein viewer interactively ties various parts of the annotation page. Selecting a predicted domain or other feature in any of the data tables will automatically highlight its position in the protein. Since many predicted features are not directly displayed in the protein schematic (mostly due to overlaps), this function simplifies the visual identification of relations among different protein features.

If a more fine-grained evaluation of a protein region is required, the viewer allows interactive selection of various parts of the protein sequence independent of the annotated features, and their submission to BLAST analysis.

Detailed information about any detected protein feature can be displayed in streamlined floating popup dialogs, enhancing the user experience and lowering the need to navigate across different web pages. A condensed version of domain annotation pages is included in the dialogs, with optional links to the complete annotation. In addition, several convenience functions are included, allowing users to copy the underlying amino acid sequence to their clipboard, or to submit the feature subsequence for further BLAST analysis.

## EXPANDED PROTEIN INTERACTION DATA

With the update of the underlying protein databases, we have also synchronized our protein interaction data with the version 11 of the STRING database (4). Updated graphical representations of putative interaction partners are now available for >21 million proteins, with direct links to the corresponding network display pages in STRING where users can explore the interaction networks in detail.

## UPDATED TAXONOMIC TREE DATA EXPORT

Domain architecture analysis functions in SMART allow users to simply access proteins containing combinations of particular domains. These can be also generated using combinations of GO terms associated to protein domains, and restricted to various taxonomic classes. In addition to the standard SMART protein schematic visualization, these data can also be exported into FASTA files or phylogenetic trees. The phylogenetic tree export has been updated and made compatible with the version 5 of the Interactive Tree of Life (iTOL) (15), with which these trees and their associated protein domain datasets are visualized, and can also be further annotated. Furthermore, the taxonomic database used for the tree generation was synchronized with the current NCBI taxonomy release (16).

## DATABASE AND WEB SERVER OPTIMIZATIONS

The backend of SMART is a relational database management system (RDBMS), powered by the PostgreSQL engine, which stores the annotation of all SMART domains, protein annotation and sequences, taxonomy information and the pre-calculated protein analyses for the entire Uniprot (2), Ensembl (3) and STRING (4) proteomes. In addition to the predictions of all SMART and Pfam (12) domains, this includes various protein intrinsic features, like signal peptides, transmembrane and coiled coil regions. Due to constant growth of the number of annotated features, we are regularly restructuring our backend databases, and optimizing various parts of the server code in order to make the user experience satisfactory. Additionally, the server hardware that powers the sequence annotation searches and database queries has been replaced and significantly expanded with additional RAM and CPUs, greatly increasing the processing speed of user submitted proteins, and lowering the overall response times.

## DATA AVAILABILITY

SMART data is freely available to academic users through EMBLem (www.embl-em.de).
